# Study on Photon Transport Problem Based on the Platform of Molecular Optical Simulation Environment

**DOI:** 10.1155/2010/913434

**Published:** 2010-04-22

**Authors:** Kuan Peng, Xinbo Gao, Jimin Liang, Xiaochao Qu, Nunu Ren, Xueli Chen, Bin Ma, Jie Tian

**Affiliations:** ^1^School of Electronic Engineering, Xidian University, Xi'an, Shaanxi 710071, China; ^2^Life Sciences Research Center, School of Life Sciences and Technology, Xidian University, Xi'an, Shaanxi 710071, China; ^3^Institute of Automation, Chinese Academy of Sciences, Beijing 100190, China

## Abstract

As an important molecular imaging modality, optical imaging has attracted increasing attention in the recent years. Since the physical experiment is usually complicated and expensive, research methods based on simulation platforms have obtained extensive attention. We developed a simulation platform named Molecular Optical Simulation Environment (MOSE) to simulate photon transport in both biological tissues and free space for optical imaging based on noncontact measurement. In this platform, Monte Carlo (MC) method and the hybrid radiosity-radiance theorem are used to simulate photon transport in biological tissues and free space, respectively, so both contact and noncontact measurement modes of optical imaging can be simulated properly. In addition, a parallelization strategy for MC method is employed to improve the computational efficiency. In this paper, we study the photon transport problems in both biological tissues and free space using MOSE. The results are compared with Tracepro, simplified spherical harmonics method (*S* *P*_*n*_), and physical measurement to verify the performance of our study method on both accuracy and efficiency.

## 1. Introduction

Optical imaging has become a research focus over the past years for its high sensitivity, nonionizing radiation, and high cost-effectiveness [1, 2]. In optical imaging, the photons escaping from the organism surface are registered at a high sensitivity charge-couple device (CCD), which can be analyzed to provide information on an organism's physiological processes. Physical experiment and numerical simulation are two common methods to study optical imaging processes. Physical experiment produces reliable results. However, the experimental preparation and operation are usually complicated and time-consuming. In addition, the scientific-grade CCD camera needed by optical imaging is relatively expensive. Because of its low-cost, simplicity and acceptable accuracy, the numerical simulation has been widely used in the field of optical imaging [3–10]. 

The key problem of numerical simulation research for optical imaging is how to simulate photon transport in various mediums. Photon transport in biological tissues can be accurately described by the radiative transport equation (RTE), which is an approximation to the Maxwell equations [4, 11]. Deterministic and statistical techniques are two common approaches for the solution of RTE. Deterministic techniques are not mature enough yet, especially for the case of highly absorbing medium. Statistic techniques, such as Monte Carlo (MC) method, can solve RTE accurately by sampling a mass of random variables relevant to the physical processes. Since introduced by Wilson and Adam in 1983 [10], the MC method has become a standard method of simulating photon transport in turbid mediums for its excellent performance [3, 4]. Recently, some simulation software or codes have been developed based on the MC method, including MCNP [12], MCML [6], TriMC3D [7], TracePro (Lambda Research Corporation, Colorado, USA.), and tMCimg [8]. Although these software or codes can be applied to the simulation of photon propagation in turbid media, they all encounter some limits in optical imaging. MCNP cannot deal with the irregular shape of biological tissues. Furthermore, it is complicated and difficult for general user to access due to its specific application in nuclear physics. MCML is a specific code for photon transport simulation in turbid media. However, it is limited to the simple multilayered medium and external light sources. TriMC3D is a source code without user interface, so an additional program has to be written to use it. TracePro is software developed for the designing and analysis of optical or illumination system, it cannot provide the photon absorption information inside the tissues. Moreover, it provides poor simulation efficiency. Similar to MCML, tMCimg can only simulate the collimated light source outside the tissues. In order to better simulate photon transport in optical imaging, we have developed a simulation platform named Molecular Optical Simulation Environment (MOSE) [9]. It has several significant features. Firstly, it simulates steady isotropic light source with arbitrary shape inside the tissues. Secondly, it provides various simulation information, including the photon absorption and transmittance information for each spectral band. Thirdly, it employs the structure information acquired by CT or MRI to describe the irregular shape and complex structure of biological tissues. Fourthly, a visual user interface is provided to ease the operation. Lastly, two new features have been added recently: the parallelization strategy for MC simulation and a free space photon transport model based on hybrid radiosity-radiance theorem [13, 14]. These new features significantly improve the simulation efficiency of MC and the simulation quality of noncontact measurement.

The reminder of this paper is organized as follows. First, the parallel accelerated Monte Carlo method for photon transport in biological tissues is introduced in Section 2. The hybrid radiosity-radiance theorem for photon transport in free space is presented in Section 3. In Section 4, the performance of our study method is verified by numerical simulation and physical experiment. The discussion and conclusion are present in Section 5.

## 2. Parallel Accelerated Monte Carlo Method for Photon Transport in Biological Tissue

MC method relies on repeated sampling of random variables to calculate the results. When it is applied to physics, a random model is first constructed in the way that each random variable obeys the statistical distribution of a physical quantity. Then plenty of samples for these random variables are taken to provide interesting results [15]. 

MC is well acknowledged to be naturally parallel, so the parallelization strategy could be a powerful tool to improve its efficiency. The parallelization of MC is virtually to parallelize the pseudorandom generator. In MOSE platform, we parallelize our pseudorandom generator by dividing a random number sequence equally into several subsequences according to the number of processor in a parallel computer system. Each processor only uses the random number from its relevant subsequence. Moreover, random number seed is used to decrease the communication between processors: only the first random number of each sub-sequence is sent to the corresponding processor as a random number seed. With these random number seeds, each processor can generate the relevant random number sub-sequence by itself.

It is the light power distributions rather than energy distributions in biological tissue that are concerned by us in practical application, so the photon is replaced by abstract photon packet which represents the light power in our MC algorithm. The power of each photon packet is calculated by dividing the total power of the light source by the number of photon packets. The photon packet transport in biological tissues consists of three major parts: generation, movement, and interactions with tissues.

**Figure 1:**
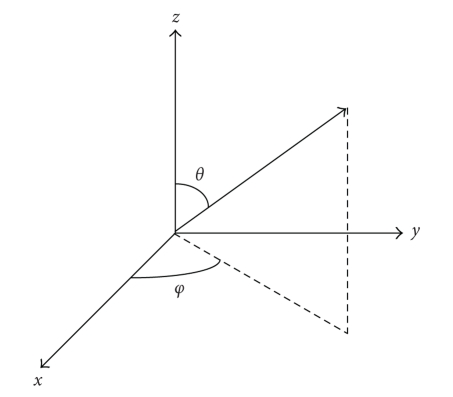
The schematic diagram of azimuth *φ* and inclination *θ*.

When a photon packet is generating by light source, its initial position and movement direction are decided by the sampling operation. Under the assumption of steady isotropic light source, the initial position can be calculated as [16]

(1) $$\;\begin{array}{c} x=x_{\min\;}+\left(x_{\max\;}-x_{\min\;}\right)\xi_{1},\\ y=y_{\min\;}+\left(y_{\max\;}-y_{\min\;}\right)\xi_{2},\\ z=z_{\min\;}+\left(z_{\max\;}-z_{\min\;}\right)\xi_{3}, \end{array}$$

where the subscripts min and max represent the lower and upper bounds of light source coordinate range, *ξ*_*i*_ (*i* = 1,2, 3) are three uniform unit random numbers. If the generated initial position does not locate inside the light source, it will be discarded and generated again. The initial movement direction can be obtained as [16]

(2) $$\;\begin{array}{c} \varphi =2\pi \xi_{\varphi },\\ \theta =\text{arccos}\;\left(2\xi_{\theta }-1\right), \end{array}$$

where *φ* and *θ* are azimuth and inclination as shown in Figure 1, *ξ*_*φ*_ and *ξ*_*θ*_ are two uniform unit random numbers, which means they are distributed uniformly over [0, 1]. 

Photon packet moves a short distance between its two interactions with tissues. The movement direction depends on its initial movement direction or its last interaction with tissue. The movement length *s* is defined by free path whose probability density function is [17]

(3) $$p\left(s\right)=\left(u_{a}+u_{s}\right)e^{-(u_{a}+u_{s})s},$$

where *μ*_*a*_ and *μ*_*s*_ are absorption coefficient and scattering coefficient of biological tissue. By sampling (3), we can calculate the free path as follows [16]:

(4) $$s=\frac{-\ln\;\xi }{\left(\mu_{a}+\mu_{s}\right)},$$

where *ξ* is a uniform unit random number.

Photon packet's interaction with tissues is including absorption, scattering, boundary effect, and termination. If a photon packet finishes one free path without hitting the tissue boundary, absorption and scattering happen. As a result of absorption, the photon packet will lose some of its power which is defined by [17]

(5) $$\;\Delta W=\frac{\mu_{a}W}{\left(\mu_{a}+\mu_{s}\right)},$$

where *W* is the power of photon packet before this absorption. The lost power will be recorded in the absorption matrix whose element is related to the power absorption at a specific position in tissues. 

Furthermore, the transport direction of photon packet is changed by the scattering as following: letting the transport direction before this scattering be *Z* axis, the new transport direction can be defined by an azimuth *φ* and an inclination *θ*. The inclination represents the angle between new and old transport directions, and it is determined by the scattering phase function. According to the Henyey-Greenstein phase function [18] which is employed in our MC algorithm, the inclination is defined by [16]

(6) $$\cos\;\theta =\{\begin{array}{cc} \frac{1}{2g}\left(1-g^{2}-{\left(\frac{1-g^{2}}{1-g+2g\xi_{\theta }}\right)}^{2}\right) & \text{if}\;g\neq 0,\\ 2\xi_{\theta }-1, & \text{others}. \end{array}$$

The azimuth *φ* is uniformly distributed over the interval (0, 2*π*), so we can easily get *φ* = 2*π* *ξ*_*φ*_, where *ξ*_*φ*_ and *ξ*_*θ*_ are two uniform unit random numbers. 

**Figure 2:**
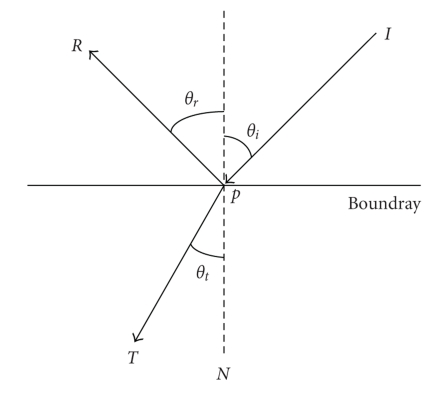
The schematic diagram of boundary condition.

When a photon packet moves from a tissue of refractive index  *n*_*i*_ into another tissue with refractive index *n*_*t*_, the boundary effect shown in Figure 2, must be considered. If the incident angle satisfies *θ*_*i*_ < *θ*_*c*_, both reflection and transmission will happen; otherwise the photon packet will only be reflected. Herein *θ*_*c*_ is the critical angle which depends on *n*_*i*_ and *n*_*t*_ as [17]

(7) $$\theta_{c}=\{\begin{array}{cc} \arcsin\left(\frac{n_{t}}{n_{i}}\right) & \text{if}\;n_{i}>n_{t},\\ 0, & \text{others}. \end{array}$$

According to the Fresnel equation, the ratio between reflection power and transmission power is determined by [16]

(8) $$R\left(\theta_{i}\right)=\{\begin{array}{cc} \frac{1}{2}\left(\frac{\text{si}{\text{n}}^{2}\left(\theta_{i}-\theta_{t}\right)}{\text{si}{\text{n}}^{2}\left(\theta_{i}+\theta_{t}\right)}+\frac{\text{ta}{\text{n}}^{2}\left(\theta_{i}-\theta_{t}\right)}{\text{ta}{\text{n}}^{2}\left(\theta_{i}+\theta_{t}\right)}\right) & \text{if}\;\theta_{i}\leq \theta_{c},\\ 1, & \text{others}, \end{array}$$

where *θ*_*t*_ is the transmission angle. If both reflection and transmission happen, one photon packet will be divided into two parts. That could worsen the computational efficient significantly. So an approximation is employed here: generating a uniform unit random number *ξ*, if *ξ* ≤ *R*(*θ*_*i*_), photon packet will be reflected totally, otherwise transmitted totally. This approximation will approach the exact solution if enough photon packets are simulated. According geometrical optics, the reflection angle *θ*_*r*_ equals the incident angle *θ*_*t*_, and the transmission angle can be solved from Snell law which is defined by

(9) $$n_{i}\sin\theta_{i}=n_{t}\sin\theta_{t}.$$

The reflection direction vector *R* and transmission direction vector *T* can be easily decided by the incident unit vector *I* and boundary normal unit vector *N* at incident point *P* as [16]

(10) $$\;\begin{array}{c} R=I-2\left(I\cdot N\right)N,\\ T=\sin\theta_{t}\frac{I-\left(I\cdot N\right)N}{\left|I-\left(I\cdot N\right)N\right|}+\text{SIGN}\left(I\cdot N\right)\cos\;\theta_{t}N, \end{array}$$

where SIGN(·) is the sign function.

The photon packet transport in tissues can be terminated under one of the following two conditions: escaping from the organism or being absorbed completely. When a photon packet escapes from the organism, its residual power is recorded in a transmission matrix whose element is related to the power transmission at a specific position on organism surface. When the power of a photon packet is less than a predetermined threshold value, a “Russian roulette” technique [10] will be used to decide its fate. This technique gives the photon packet one chance in *m* (e.g., *m* = 10) to continue its transport with an amplified weight which is defined by

(11) $$\;W=\{\begin{array}{cc} mW & \text{if}\;\xi \leq \frac{1}{m},\\ 0, & \text{others}, \end{array}$$

where *ξ* is a uniform unit random number. 

## 3. Hybrid Radiosity-Radiance Theorem for Free Space Photon Transfer

Photons escaped from biological tissue surface will transport in free space where no absorption and scattering effects exist but camera lens effects become important. A free space photon propagation model has been proposed by Ripoll [13], who firstly presents and demonstrates the possibility of realizing qualitative noncontact optical tomography. A model based on hybrid radiosity-radiance theorem has been introduced by Chen recently [14], which takes a complicated optical system into account, including optical layout and objective effects analysis such as perspective effects, image aberrations, and depth-of-field effects. The simulated detector result obtained by the latter is similar to the physical measurement, so we use it as the simulation algorithm for the photon progress in free space. Once the organism outside surface flux density *J*_*n*_(*r*) [W/mm^2^] has been obtained by MC method, photon transport model in free space can be modeled as the follows. A differential surface area *d* *S* of unit normal vector *N*_*s*_ centered at *r* constitutes a new light source named Lambertian source for the differential detector area *d* *A* of unit normal vector *N*_*d*_ centered at *r*_*d*_ [14]. It would act as a radiation source and irradiate to the surrounding space isotropically [19], which means that its radiance is constant and independent of the solid angle but varies in different position. The Lambertian source can be characterized by the radiance *L*(*r*) [W/mm^2^ *sr*] which is defined by the flux per unit area per unit solid angle. Therefore, the following relationship between the surface flux density *J*_*n*_(*r*) and radiance *L*(*r*) of a differential surface area *d* *S* centered at *r* can be derived as [19]

(12) $$L\left(r\right)=\frac{1}{\pi }J_{n}\left(r\right).$$

Based on the inverse square law of distance, which depicts the relationship between radiant intensity of a point source or a microsurface source and irradiance irradiated by the source, the microunit power of a differential detector area *d* *A* centered at *r*_*d*_ received from a differential surface area *d* *S* centered at *r* is [19]

(13) $$dP\left(r_{d}\right)=I\left(r\right)\frac{s_{r-r_{d}}\cdot dA}{{\left|r-r_{d}\right|}^{2}},$$

where *s*_*r*−*r*_*d*__ is a unit vector denoting the direction from *r*_*d*_ pointing to *r*; *I*(*r*) [*W/sr*] is the specific intensity at the surface point *r* of differential area *d* *s* and can be calculated using the following formula [19]

(14) $$I\left(r\right)=L\left(r\right)\left[dS\cdot s_{r_{d}-r}\right],$$

where *s*_*r*_*d*_−*r*_ is a unit vector denoting the direction from *r* to *r*_*d*_. Substituting (12) and (14) into (13), we can obtain the following expression:

(15) $$\;dP\left(r_{d}\right)=\frac{1}{\pi }J_{n}\left(r\right)\frac{\left[s_{r_{d}-r}\cdot dS\right]\left[s_{r-r_{d}}\cdot dA\right]}{{\left|r_{d}-r\right|}^{2}}.$$

Equation (13) can be conveniently rewritten as

(16) $$dP\left(r_{d}\right)=\frac{1}{\pi }J_{n}\left(\vec{r}\right)\frac{\left[\cos\;\theta_{s}\cos\;\theta_{d}dSdA\right]}{{\left|r_{d}-r\right|}^{2}},$$

where cos *θ*_*s*_ = *s*_*r*_*d*_−*r*_ · *N*_*s*_ is the cosine dependence of the Lambert's law, cos *θ*_*d*_ = *s*_*r*−*r*_*d*__ · *N*_*d*_ accounts for the detector orientation *N*_*d*_ with respect to the line sight; *d* *S* and *d* *A* are the area of the differential surface and detector unit, respectively.

Integrating equation (16) over all the surface points that are visible from the lens system and taking into account the influence of the lens system, we obtain the total photon flux at *r*_*d*_ as [14]

(17) $$P\left(r_{d}\right)=\frac{1}{\pi }\int_{s}J_{n}\left(r\right)\xi \left(r,r_{d};f\right)\frac{\left[\cos\;\theta_{s}\cos\;\theta_{d}dA\right]}{t^{2}{\left|r_{d}-r-\left(tu^{2}/f\cos\;\theta \right)s\right|}^{2}}dS,$$

where *ξ*(*r*, *r*_*d*_; *f*) is a visibility factor that discards the surface points invisible from the lens and depends mainly on the parameters of the lens system configured in the optical system; *t* is the magnification ratio of the lens system and can be calculated through *t* = *v*/*u*; object distance *u* can be calculated using the lens law when the object distance is determined; *f* is the focus of the lens system; *s* is a unit vector along the line of sight; *θ* is the angle between the line of sight and optical axis.

## 4. Experiments and Results

In order to evaluate the performance of our study method, we perform both numerical simulations and physical experiments. In the numerical simulations, phantoms and digital mouse are designed to verify the performance of our method on photon transport in biological tissues. The MOSE simulated surface flux density is compared with that of Tracepro (Version 3.2.2 release) and simplified spherical harmonics method [20]. Furthermore, the effect of parallelization is also verified by the comparison of time cost between parallel and serial MC method. In physical experiment, a cylindrical phantom is utilized to validate the performance of our method on non-contact measurement. In following experiments, the normalized root mean square error (NRMSE) $\overset{̅}{e}$ is used to estimate the discrepancy between two normalized data *d* ^1^ and *d* ^2^. The NRMSE is defined as

(18) $$\overset{̅}{e}\left(d^{1},d^{2}\right)=\sqrt{\frac{1}{N}\overset{N}{\underset{i=1}{\sum }}{\left(d_{i}^{1}-d_{i}^{2}\right)}^{2},}$$

where *d* ^1^ = [*d*_1_^1^, *d*_2_^1^,…, *d*_*N*_^1^], *d* ^2^ = [*d*_1_^2^, *d*_2_^2^,…, *d*_*N*_^2^], *N* is the dimension of data.

### 4.1. Homogeneous Numerical Phantom Experiment

**Figure 3:**
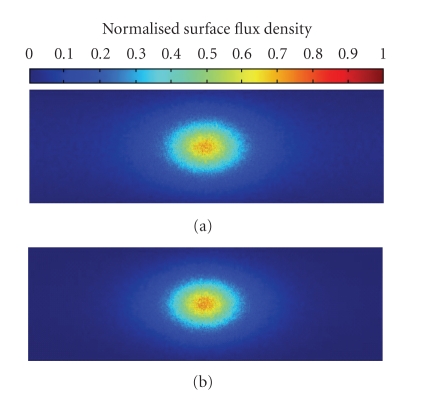
Normalized side surface flux density of MOSE and Tracepro. (a) Results of MOSE; (b) results of Tracepro.

**Figure 4:**
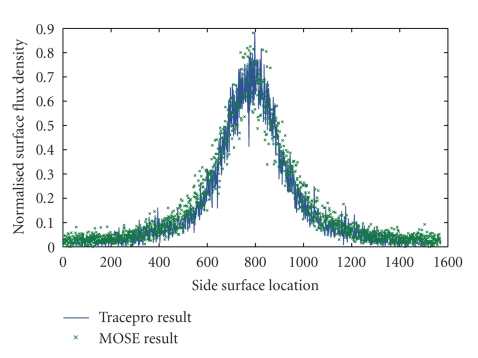
Comparison of side surface flux density between MOSE and Tracepro at the position *z* = 0 mm.

A cylindrical homogeneous numerical phantom of 15 mm radius and 30 mm height is used in this experiment to test our method's performance with the regular shaped homogeneous object. The phantom's center is located at (0, 0, 0) mm. The priori optical parameters according to [21] are specified as absorption coefficient *μ*_*a*_ = 0.0138 mm^−1^ and reduced scattering coefficient *μ*_*s*_′ = (1 − *g*)*μ*_*s*_ = 0.91 mm^−1^. An internal cylindrical light source of 1 mm radius and 2 mm height is centered at (8, 0, 0) mm, the power of which is 1 nW. This phantom is simulated by MOSE platform with the simulated photon packets number of 10^6^. The simulated flux density on the cylinder side surface is mapped into a 2D image with the resolution of 500∗1570. Because the size of phantom side surface is 30∗94.2 mm, each pixel in the image is responding to an area of 0.06∗0.06 mm on the phantom side surface. The MOSE simulation results are shown in Figure 3(a). The Tracepro result is present in Figure 3(b). Comparing two results, it can be found that MOSE produces good agreement with Tracepro. Moreover, the data at the position *z* = 0 mm are taken out from two results to do further comparison in Figure 4. The NRMSE between these two curves is 1.71%, and we expect the NRMSE to decrease further if more photon packets are simulated. Additionally, with the same quantity of simulated photon packet, the simulation time of MOSE is about 12 minutes, which is much shorter than 3.3 hours cost by Tracepro. 

### 4.2. Digital Mouse Experiment

**Figure 5:**
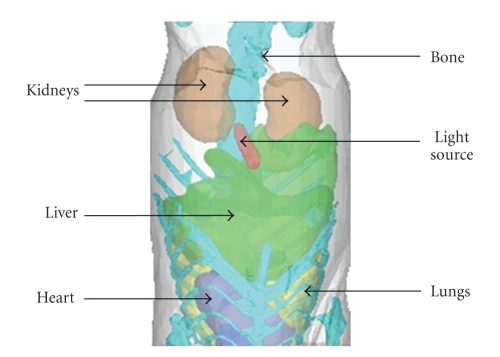
Tissue geometrical information of the digital mouse obtained by micro-CT.

**Figure 6:**
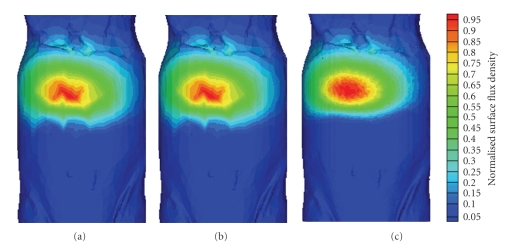
Normalized surface flux density based on digital mouse. (a) MOSE results with coarse mesh; (b) *S* *P*_3_ result with coarse mesh; (c) MOSE results with fine mesh.

In this experiment, the structure information of a mouse, which is present in Figure 5, is obtained by a micro-CT system. Then, a simulation is carried out to test the ability of our method in dealing with object that has irregular shape and complicated structure. Six types of tissues are included in the digital mouse, that is, fat, heart, lung, liver, kidney, and bone, as shown in Figure 5. The optical parameters according to [22] are listed in Table 1. A capsule filled with the compound solution from a luminescent mini glow light stick is implant below the liver as a light source. Recently, the simplified spherical harmonics (*S* *P*_*n*_ *n* = 3,5, 7,…) equation, which is a second-order approximation form of the RTE, has been developed for optical imaging. It can get more accurate results than DE, especially for the case of high-absorbing media [20]. In this paper, the *S* *P*_3_ method, which solves the *S* *P*_3_ equation by finite element method, is used to verify the accuracy of MOSE. The digital mouse is discretized by a volume mesh for the finite element method, and this mesh is composed of 21154 nodes and 592676 triangle elements. However, only 8670 of 21154 nodes and 33205 of 592676 triangles elements, which are used to construct the surfaces of each tissue, are needed by MOSE. That means that MOSE can achieve the same surface resolution with much less nodes and elements than *S* *P*_3_. The photon packages number simulated by MOSE is 10^8^. The normalized surface flux density obtained by MOSE is shown in Figure 6(a). Comparing with the *S* *P*_3_ simulation results present in Figure 6(b), it can be found that two methods get quite similar results. Both the flux density distribution of MOSE and *S* *P*_3_ results are not very smooth. This problem is caused by the low surface mesh resolution used here, and it can be improved by using a finer mesh to discretize the digital mouse. With the tissue surfaces taken from a fine mesh discretization which is consisting of 355469 nodes and 10308601 triangles, a much more smooth simulation result can be obtained by MOSE, as present in Figure 6(c). However, it is difficult to employ this finer mesh to *S* *P*_3_ method, because the memory needed by *S* *P*_3_ method increases fast with the number of nodes in the mesh. The memory requested by *S* *P*_3_ method with the coarse mesh in this experiment already reaches 15 GB. However, the memory consumption of MOSE is less than 300 MB even with the fine mesh. This is because the nodes and elements needed by MOSE are much less than *S* *P*_3_ if the same mesh resolution is used. More importantly, the huge coefficient matrix (the dimension is two times of node number), which is need to be constructed and processed in *S* *P*_3_ method, can be totally avoid in MOSE. So we find MOSE to be more suitable to simulate the photon transport in biological tissues than *S* *P*_3_ method, because massive nodes and elements are usually needed to approximate the irregular shape and complex structure of biological tissues. 

### 4.3. Parallelization Experiment

**Figure 7:**
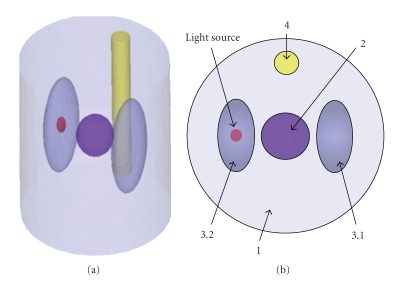
Numerical phantom for parallelization experiment and its cross-section. (a) Numerical phantom; (b) cross-section of (a).

A small parallelization computational system based on several LAN-linked PCs (Intel Core 2 CPU 6550 @ 2.33 GHz and 2 GB RAM) is employed to evaluate the acceleration performance of the parallelization strategy in MOSE. A heterogeneous numerical phantom, whose optical parameters are specified as Table 2, is used in this experiment, as depicted in Figure 7. An ellipsoid light source with radiuses of 0.5 mm, 0.5 mm, and 1 mm is located at (15, 40, 12) mm, and its power is 1 nW. With the simulated photon packet number of 10^8^, several simulation experiments are performed with 1, 2, 4, 6, and 8 processors to acquire the phantom surface flux density. Table 3 presents the simulation time of each experiment and the acceleration effect of parallelization strategy. The results indicate that the time cost of MOSE can be reduced significantly by parallelization strategy. However, because the data amount of calculation results produced by one processor in parallelization strategy is equal to that in the serial strategy, the more processors we used, the larger amount of result data is needed to be transmitted to the host processor to construct the final results at the end of the simulation. Since these data transmissions can only be performed serially, the acceleration efficiency is decreased with the increasing of processor number due to the extra time spent on the interprocessor data transmissions.

### 4.4. Physical Experiment

**Figure 8:**
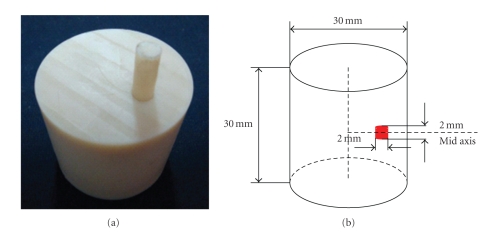
Cylinder physical phantom. (a) Physical phantom with one light source; (b) schematic diagram of numerical calculation phantom.

**Figure 9:**
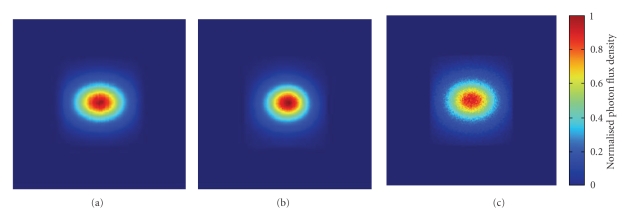
Normalized detection flux density of simulation and physical experiment. (a) Results of MOSE; (b) results of physical measurement; (c) results of MC method.

**Figure 10:**
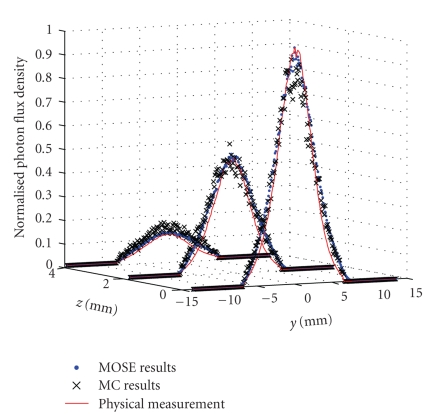
Comparison between the simulation and physical measured detection flux density at the positions *z* = 0 mm, *z* = 2 mm, and *z* = 4 mm.

A nylon cylindrical homogeneous physical phantom is used to evaluate the performance of our method on non-contact measurement, as shown in Figure 8. The phantom's radius and height are 15 mm and 30 mm, respectively, and its center is located at (0, 0, 0) mm. The phantom's optical parameters, which are measured at the wavelength about 660 nm by a time-correlated single photon counting system [21], are listed as absorption coefficient *μ*_*a*_ = 0.0138 mm^−1^ and reduced scattering coefficient *μ*_*s*_′ = (1 − *g*)*μ*_*s*_ = 0.91 mm^−1^. The compound solution from a luminescent mini glow light stick (Glowproducts, Victoria, Canada) is injected in a cylindrical hole of 1 mm radius in the phantom as the light source. The center of the light source is located at (8, 0, 0) mm, and its height is 2 mm. The light emitted by the compound solution has the wavelength round 660 nm. A PIXIS 2048B CCD camera, which is coupled with an optical lens subsystem (Nikon Nikkor Micro) of 55 mm focal length, is used to register the photons escaped from phantom surface. The detector is located at (256, 0, 0) mm, and its size is 16∗16 mm. MOSE simulation results and physical measurement are shown in Figures 9(a)-9(b). Comparing with the MC method simulation results (the simulated photon packet number is 10^9^) present in Figure 9(c), it can be found that MOSE simulation results are more smooth, which makes it more close to the physical measurement. Furthermore, MOSE simulation results get better data distribution agreement with physical measurement in the central area of the light spot. The data at three positions *z* = 0 mm, *z* = 2 mm, and *z* = 4 mm are taken out from three results to do further examination, as shown in Figure 10. Although three results have similar trend, MOSE obviously gives better results than MC method round the peaks of *z* = 0 curve and *z* = 4 curve, which means that the MC results are not as accurate as the MOSE results at the central and boundary area of light spot. The NRMSE between MOSE results and physical measurement of each curve can be calculated as 3.99%, 3.02%, and 1.05%, respectively. They are smaller than the NRMSE for MC method which are 5.20%, 3.98%, and 2.21%. 

## 5. Discussion and Conclusion

Although the parallelization strategy can improve the efficiency of MC method significantly, the CPU-based parallelization computational system employed in this paper is difficult to be constructed and applied. Our future work will focus on the parallelization strategy for MC method based on graphics processing units (GPUs) which will offer large performance benefits with a single graphic card. 

Although MOSE produces better results than MC method in the photon transport in free space, some differences between the MOSE simulation results and physical measurement can still be observed in Figures 8 and 9. Firstly, the MOSE simulation results are slightly discontinuous, which is caused by the low resolution of phantom surface flux density data. Unfortunately, although the surface data with better resolution can be produced easily by MOSE, the low execution efficiency of free space photon transport algorithm prevents the utility of it. This problem may be solved by code optimization and GPU parallelization in our future work. Secondly, the MOSE results are slightly fuzzy than physical measurement, which makes the light spot look larger than the physical measurement. Through further experiment, we have found that it is caused by the influence of aperture which is not considered by the algorithm mentioned in this paper. An improved algorithm is already under development, the results will be reported lately.

In conclusion, the study for photon transport in optical imaging is carried out based on the MOSE simulation platform. As a standard method, MC method is employed to simulate photon transport in biological tissues, and its time cost is decreased significantly by the parallelization strategy. The photon transport in free space is simulated based on the hybrid radiosity-radiance theorem which is combined with the effects of lens system, so the non-contact measurement, which is a very important detecting mode in optical imaging, can be simulated properly. The performance of our study method is demonstrated by numerical simulations and physical experiments. 

MOSE can be downloaded freely from http://www.mosetm.net/.

## Figures and Tables

**Table 1:** Optical parameters of the digital mouse.

Tissue	*μ*_*a*_ (mm^−1^)	*μ*_*s*_′ (mm^−1^)
Fat	0.0057	1.2374
Heart	0.0910	1.0291
Lung	0.3045	2.2273
Liver	0.5458	0.7115
Kidney	0.1021	2.4144
Bone	0.0943	2.6691

**Table 2:** Geometrical information and optical parameters of the phantom in parallelization experiment.

Tissue index	Center (mm)	Geometrical information (mm)	*μ*_*a*_ (mm^−1^)	*μ*_*s*_ (mm^−1^)	*g*
1	(0, 0, 0)	cylinder, radius 8, height 20	0.01	4	0.90
2	(0, 0, 0)	ellipsoid, radius: 4, 4, 5	0.2	16	0.85
3.1	(0, −4, 0)	ellipsoid, radius: 6, 3, 12	0.35	23	0.94
3.2	(0, 4, 0)	ellipsoid, radius: 6, 3, 12	0.35	23	0.94
4	(0, 6, 0)	cylinder, radius 1, height 18	0.002	20	0.90

**Table 3:** Time consumption of MOSE in parallelization experiment.

Number of processor	1	2	4	6	8
simulation time cost (s)	31784	15992	8148	5424	4231
acceleration ratio	1	1.9875	3.9008	5.8599	7.5122
